# Fear of cancer occurrence in breast cancer previvors: a scoping review of the literature

**DOI:** 10.3389/fpsyg.2026.1887732

**Published:** 2026-08-11

**Authors:** Christa Torrisi, Jeanne Ward, Victoria Champion

**Affiliations:** Indiana University School of Nursing, Indianapolis, IN, United States

**Keywords:** BRCA, breast cancer, fear of cancer, pathogenic variant, previvor

## Abstract

**Objective:**

Pathogenic variants (PVs) in genes create a unique population of “previvors,” individuals who have no personal history of a cancer diagnosis, but carry chronically elevated lifetime cancer risk. PVs in breast cancer previvors negatively impact body image and family planning, and increase uncertainty and anxiety. How fear of cancer occurrence (FOCO) is experienced in previvors is not well described in the literature. This scoping review aimed to describe how FOCO manifests among breast cancer previvors.

**Methods:**

Guided by Joanna Briggs Institute (JBI) scoping review methodology, a search was conducted across CINAHL, PsycINFO, PubMed, and Scopus databases. Records were screened and data extracted using Covidence. Inclusion criteria were female sex, presence of a breast cancer PV, no personal history of breast cancer, and description(s) of cancer-related fear.

**Results:**

1,022 records were screened, 10 articles (*n* = 620 previvors) met inclusion criteria. FOCO manifested among breast cancer previvors in three ways, (1) perception of cancer occurrence, particularly if a cancer diagnosis was perceived by the previvor as inevitable; (2) misinterpretation of information from clinician communications, websites, or somatic sensations; and (3) heightened “scanxiety” of a cancer diagnosis during routine breast screening.

**Conclusion:**

FOCO may represent a distinct and clinically important experience to individuals living with an increased predisposition to developing breast cancer. Previvors must navigate both present and future cancer fears, distinguishing them from both average-risk individuals and cancer survivors. However, more research is needed to better characterize and measure FOCO among individuals with an increased risk of developing breast cancer.

## Introduction

Approximately 5–10% of breast cancers result from an inherited pathogenic variant (PV) (American Cancer Society, 2026), a change in the DNA sequence of a gene that causes a person to be at risk of genetic disease, such as cancer (National Cancer Institute, 2026). With over 320,000 new breast cancer diagnoses estimated for U. S. women in 2026 (American Cancer Society, 2026), up to 32,000 of these cases may have a hereditary etiology. The genetic link of breast cancer among family members was first established in the early 1990s with the discovery of BReast CAncer (BRCA) 1 and 2 PVs (Miki et al., 1994; Williams-Jones, 2002; Wooster et al., 1994). While the lifetime risk of breast cancer in women is approximately 13% (American Cancer Society, 2026), the presence of a PV significantly escalates this risk. BRCA1 and BRCA2 PVs are associated with a lifetime risk exceeding 70%; other high- and moderate-penetrance PVs, such as PALB2, ATM, and CHEK2, also confer increased lifetime risks greater than 20% (Easton et al., 2015; Kuchenbaecker et al., 2017; Marabelli et al., 2016; National Comprehensive Cancer Network, 2026; Yang et al., 2020). The term “previvor” was coined in 2000 to describe individuals who have never had cancer but carry an identified PV that increases their lifetime risk of being diagnosed with cancer (Getachew-Smith et al., 2020).

Carrying a PV has been associated with anxiety, uncertainty, and worry in many breast cancer previvors, due to chronically elevated cancer risk and its impact on family planning, decision-making, and cancer risk-management strategies (Dean and Fisher, 2019; Dick et al., 2022; Glassey et al., 2018a; Isselhard et al., 2023; Rojas et al., 2019). Less clear is how fear of cancer occurrence (FOCO), fear related specifically to the possibility of developing cancer due to an identified risk, such as the presence of a PV, is described in this population. This lack of understanding demonstrates both a knowledge and conceptual gap, particularly compared to cancer-related fear in populations of breast cancer survivors, where fear is a well-established and defined construct that has been found to negatively impact mental wellbeing, utilization of healthcare services, and quality of life (Luigjes-Huizer et al., 2022; Mehnert et al., 2009; Mutsaers et al., 2016; Otto et al., 2018).

While fear is typically described as an emotional response to an immediate threat, and anxiety is characterized as an emotional response to a potential future threat (American Psychological Association, 2018; Barlow, 1991), the previvor's fear may be positioned as both present- and future-oriented due to the ongoing but uncertain nature of cancer risk. Fear also differs from the cognitive process of uncertainty, which, in the context of illness, is the insufficient processing or categorizing of a symptom or diagnosis due to an inability to predict its outcome (Mishel, 1988). Fear is similar to but distinct from the cognitive state of worry, which is the cognitive attempt to engage in problem-solving for an uncertain future event that carries the potential for negative outcomes (Borkovec et al., 1983).

Because there is no consensus on the definition of cancer-related fear in the previvor population, we defined FOCO as an emotional and/or physiological response to the chronic present and future risk of developing cancer due to an identified genetic threat. We position that fear of cancer recurrence (FCR) in breast cancer survivors is an entirely different experience from FOCO in previvors who, while possessing an increased cancer risk, have never faced a cancer diagnosis. Because a PV presents an ongoing cancer risk that persists across the lifespan until either death or a cancer diagnosis occurs, it is vital to distinguish how FOCO appears in the previvor population to prevent or manage cancer-related fear and promote emotional and physical wellbeing. While FCR has been found to contribute to hypervigilant and avoidant behaviors, which negatively impact quality of life in cancer survivors (Hall et al., 2022; Mehnert et al., 2009; Mutsaers et al., 2016; Simard et al., 2013; Thewes et al., 2016), no review exploring cancer-related fear in the breast cancer previvor population has been identified.

To address this gap, the objective of this study was to conduct a scoping review to examine the literature on how previvors with an identified PV associated with increased breast cancer risk manifest FOCO. The review results will inform both research and clinical practice on supporting individuals with an increased risk of developing breast cancer across their lifetimes.

## Methods

A scoping review provides a systematic and rigorous approach to synthesizing the literature to identify and summarize existing evidence and gaps in a given area (Rodger et al., 2024). Using the Arksey & O'Malley principled analytical framework (Arksey and O'Malley, 2005), we performed the following five steps for this review: identifying the research question; identifying relevant studies; selecting studies; charting data; and collecting, summarizing, and reporting the results. Using the Joanna Briggs Institute (JBI) scoping review methodology (Peters et al., 2020), we examined the question: “How does fear of cancer occurrence manifest among breast cancer previvors with an identified pathogenic variant?” Using our developed definition of FOCO, two doctorally prepared members of the study team (CT and JW) created a set of search terms and search strategies from existing literature. These terms examined previvors with an increased breast cancer risk, PVs which increased lifetime breast cancer risk, actions taken to manage cancer risk, and cancer-related fear (Dean and Fisher, 2019; Getachew-Smith et al., 2020; Koch, 2022; Marabelli et al., 2016; Simard et al., 2013). Search terms (see Table 1) were selected to purposely isolate FOCO from overlapping constructs (Peters et al., 2020) such as cancer-related distress, anxiety, worry, and uncertainty (Borkovec et al., 1983; Daniel-Watanabe and Fletcher, 2021; Maheu et al., 2021). By sifting out these similar terms, we were able to examine how populations of breast cancer previvors experienced the concept of FOCO, in the context of living with an increased risk of developing breast cancer.

**Table 1:** Search terms and database results September 2025–July 2026.

Search terms	Database	Results
“fear” AND “previvor”	CINAHL	3
	PsycINFO	2
	Scopus	11
	PubMed	0
“fear of cancer” AND “BRCA” AND “screening” NOT “cancer diagnosis”	CINAHL	3
	PsycINFO	155
	Scopus	289
	PubMed	5
“fear” AND “risk reducing mastectomy” AND “unaffected”	CINAHL	0
	PsycINFO	397
	Scopus	38
	PubMed	2
“fear” AND “bilateral risk reducing mastectomy”	CINAHL	21
	PsycINFO	149
	Scopus	155
	PubMed	12
“fear” AND “chemoprevention” AND “previvor”	CINAHL	0
	PsycINFO	0
	Scopus	1
	PubMed	0
“fear” AND “increased surveillance” AND “BRCA”	CINAHL	0
	PsycINFO	1
	Scopus	195
	PubMed	8

The following health- and science-focused databases were searched between September 2025–July 2026: CINAHL, PsycINFO, PubMed, and Scopus. To our knowledge, this is the first review to examine FOCO in a population of previvors. We elected to examine breast cancer previvors due to the relative abundance of literature in this population compared to previvors of other cancers. To ensure a thorough search of FOCO in the relatively limited previvor literature, no limitations were placed on research report type, length, PV type, or age. The following inclusion criteria for searches were selected: the presence of a PV prior to study start that increased breast cancer risk (i.e., completion of genetic testing), no personal history of breast cancer, and reports published in English. Reports were included if samples that contained breast cancer survivors and previvors reported results for previvors separately.

Exclusion criteria were as follows: personal history of breast cancer; previvors with increased cancer risk other than breast cancer; no identification of the PV prior to study start; male sex; and reports describing worry, anxiety, and/or uncertainty. In the instance of reports with samples of both cancer survivors and previvors, reports were excluded if results were not separated between the two groups. Additionally, individuals at high risk without an identified PV were excluded to ensure the analysis focused specifically on FOCO in relation to a definitive biological marker of cancer risk.

Records from each search were exported to (Covidence Systematic Review Software 2026), duplicate citations were removed, and titles and abstracts were reviewed to identify articles meeting criteria for full-text review. Next, we reviewed full-text versions using the same exclusion categories. For article title, abstract review, and full-text review, research reports were screened independently (CT and JW) to identify differences and to cross-validate the extracted information. For each report meeting criteria, information was extracted, including the country, year of publication, authors, sample demographics, study design, PV type, and major findings which described how FOCO was manifested by breast cancer previvors. Data were extracted from quantitative surveys and qualitative quotes and themes. Results were then summarized (see Table 2), and any differences were discussed to achieve 100% consensus.

**Table 2:** Charting table.

First author, year of publication	Study location	Purpose	Sample size	Study design	Fear of cancer findings among previvors	PV	Time since PV diagnosis	Age range of sample
Dagan and Goldblatt (2009)	Israel	To explore how asymptomatic BRCA1 or 2 carrier women gave meaning to being concurrently healthy and at high risk for breast and ovarian cancer	*N* = 17 previvors	Qualitative: phenomenology	•Fear that cancer will occur because mother had cancer •Fear that cancer diagnosis will eventually occur	BRCA1 and BRCA2	5–8 years	26–57 years
Dean (2016)	USA	To understand the ways in which BRCA-positive patients experience uncertainty about hereditary breast and ovarian cancer	*N* = 34 previvors	Qualitative: constant comparison	•As the date of a routine screening visit approached, participants described fear that clinicians would discover cancer during the visit •During a routine visit, a suspicious finding on mammogram of MRI caused fear of cancer to occur	BRCA1 and BRCA2	Not specified	27–67 years
DiMillo et al. (2013)	Canada	To understand experiences of women who tested positive for BRCA1 or BRCA2 genes	*N* = 6 previvors	Qualitative: Grounded theory	•Ongoing fear of cancer occurring persisted after diagnosis of a BRCA PV	BRCA1 and BRCA2	At least 6 months	31–44 years
Fantini-Hauwel et al. (2025)	Belgium	To explore the significance of being a BRCA carrier by utilizing a slightly modified framework of the commonsense model	*N* = 15 (with *N* = 9 previvors, *N* = 6 survivors)	Qualitative: Interpretive phenomenological analysis	•Fear cancer is occurring when a new breast pain is experienced	BRCA1 and BRCA2	1–7 years	Entire sample: 33–67 years Previvors: 33–67 years
Glassey et al. (2018b)	Australia and New Zealand	To explore whether risk perception differed between women with a known BRCA1/2 mutation, compared to those without a known BRCA mutation	*N* = 46 (*N* = 36 BRCA previvors and *N* = 10 ‘high risk' women)	Qualitative: Interpretive phenomenological analysis	•Previvors with BRCA PVs described online resources increasing fear of cancer	BRCA1 and BRCA2 in 36 participants	Not specified	Not specified
Haroun et al. (2011)	Canada	To identify factors associated with a “late” switch from MRI-based surveillance to risk-reducing mastectomy and to measure patient satisfaction with the decision	*N* = 246 previvors	Quantitative: Prospective, using study-specific surveys	•38% of the sample listed fear of cancer, with this as a reason to undergo bilateral risk- reducing mastectomy	BRCA1 and BRCA2	Not specified	22–66 years
Hyldebrandt et al. (2025)	Norway	To investigate factors associated with the decision of when and whether to undergo RRM, help received from the health care system and whether these factors have affected satisfaction with surgery in a cohort comprised only of BRCA1/2 carriers without prior breast cancer	*N* = 272 previvors	Quantitative: Cross-section using study-specific questionnaire	•Fear of dying from breast cancer as a reason to undergo bilateral risk-reducing mastectomy •Fear that cancer would separate mothers and children	BRCA1 and BRCA2	Not specified	25–70 years
Leonarczyk and Mawn (2015)	USA and Canada	To explore the experience of cancer risk-management decision making for women who are unaffected carriers of a BRCA mutation (previvors)	*N* = 15 previvors	Qualitative: Phenomenological design	•Fear of cancer occurred after learning of the presence of a PV •Fear that cancer would separate mothers and children	BRCA1 and BRCA2	Not specified	21–63 years
Stracke et al. (2022)	Germany	To obtain a closer insight into (1) how the women perceive the situation following the disclosure of the genetic test result, (2) the personal coping strategies of women carrying PVs in moderate-risk breast cancer genes, (3) the influence of the familial medical history on the coping style, and (4) what kind of additional support the women need to cope with their life-long increased cancer risk	*N* = 12 (*N* = 3 previvors, *N* = 9 survivors)	Qualitative: Content analysis	•66% of previvors expressed fear of cancer occurring	CHEK2, RAD51C, RAD51D, PALB2, or ATM	At least 6 months	Full sample: 29–58 years Previvors: 29–38 years
Tezak et al. (2021)	USA	To assess follow-up care decision making patterns among a subset of female BRCA carriers	*N* = 27 (*N* = 8 previvors, *N* = 19 survivors)	Mixed methods	•Fear of developing breast cancer contributed to the decision to undergo bilateral risk-reducing mastectomy	BRCA1 and BRCA2	Not specified	Not specified

## Results

## General characteristics of included studies

**Figure 1:**
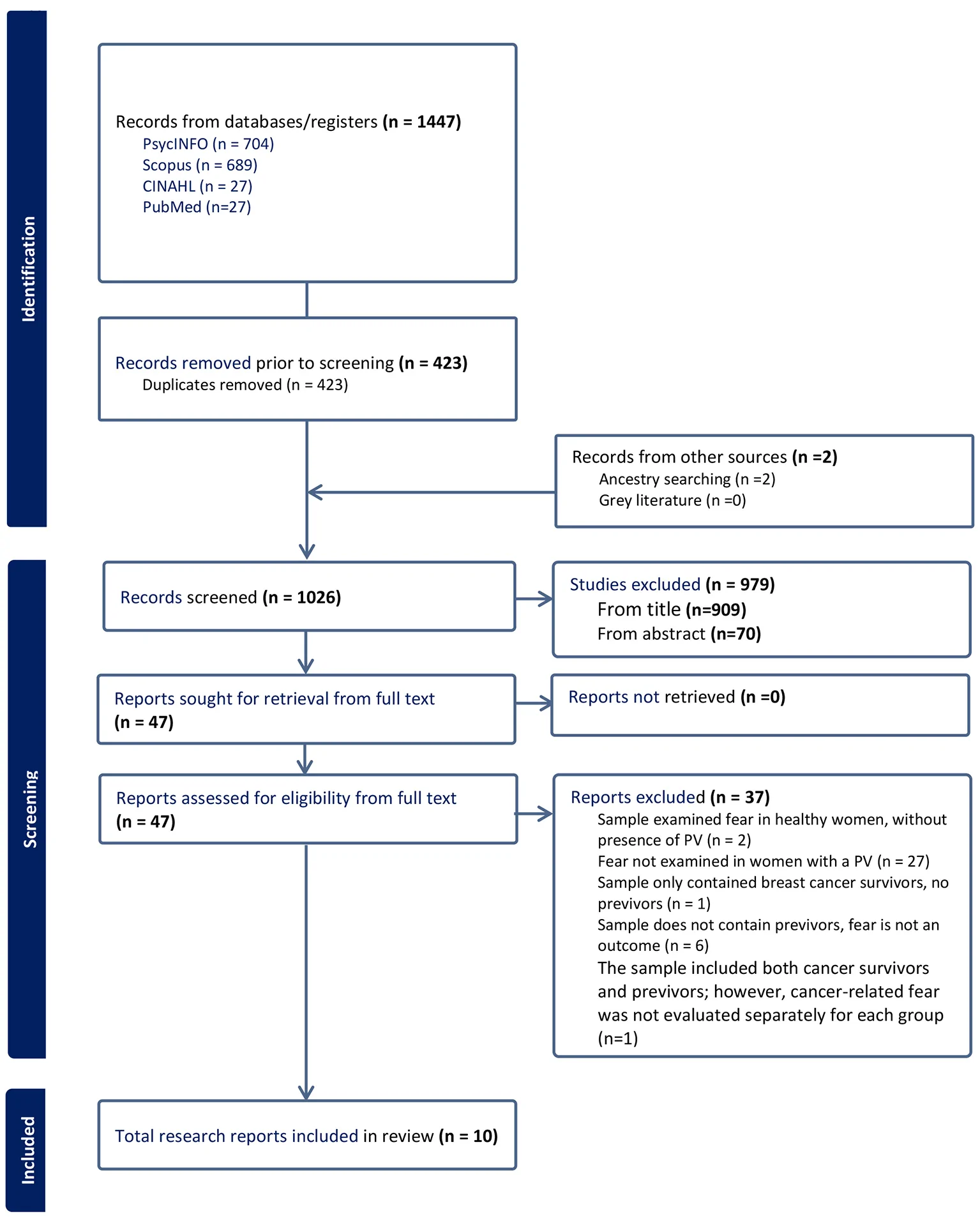
PRISMA flow diagram for article inclusion.

A total of 1,028 records were screened for inclusion in this search, and after the removal of 423 duplicates, 47 research reports qualified for full-text review. Eight research reports met the inclusion criteria for this study. An additional ancestry search of the reference lists of screened studies led to the identification and full-text review of two additional reports, both of which met the inclusion criteria. A total of 10 studies were included in this scoping review (see Figure 1).

A total of 620 previvors were included across studies. Locations included Europe (Belgium, Germany, Norway) (Fantini-Hauwel et al., 2025; Hyldebrandt et al., 2025; Stracke et al., 2022), Australia and New Zealand (Glassey et al., 2018b), the Middle East (Israel) (Dagan and Goldblatt, 2009), and North America (Canada, U. S.) (Dean, 2016; DiMillo et al., 2013; Haroun et al., 2011; Leonarczyk and Mawn, 2015; Tezak et al., 2021). Nine studies contained previvors with a BRCA PV; only one study included PVs other than BRCA1/2 (Stracke et al., 2022). Both quantitative and qualitative designs were included (see Table 2). In the two quantitative studies, which incorporated cross-sectional (Hyldebrandt et al., 2025) and prospective designs (Stracke et al., 2022), participants completed study-specific surveys incorporating questions about FOCO. Of the eight qualitative studies included in this review (Dagan and Goldblatt, 2009; Dean, 2016; DiMillo et al., 2013; Fantini-Hauwel et al., 2025; Glassey et al., 2018b; Haroun et al., 2011; Leonarczyk and Mawn, 2015; Tezak et al., 2021), all employed semi-structured interviews to obtain data; phenomenology was the most common method (Dagan and Goldblatt, 2009; Fantini-Hauwel et al., 2025; Glassey et al., 2018b; Leonarczyk and Mawn, 2015). Articles were published between 2009 and 2025, with 60% occurring in the last 10 years (Dean, 2016; Fantini-Hauwel et al., 2025; Glassey et al., 2018b; Hyldebrandt et al., 2025; Stracke et al., 2022; Tezak et al., 2021). A synthesis of the results revealed three ways in which FOCO manifests in previvors with an increased risk of developing breast cancer (see Table 3).

**Table 3:** Cross study synthesis: fear of cancer occurrence.

Manifestations of fear of cancer occurrence in breast cancer previvors	Dagan and Goldblatt (2009)	Dean (2016)	DiMillo et al. (2013)	Fantini-Hauwel et al. (2025)	Glassey et al. (2018b)	Haroun et al. (2011)	Hyldebrandt et al. (2025)	Leonarczyk and Mawn (2015)	Stracke et al. (2022)	Tezak et al. (2021)
Perceptions of cancer occurrence	X		X	X	X	X	X	X	X	X
Information misinterpretation				X	X			X	X	
Scanxiety		X	X		X					

## Perceptions of cancer occurrence

In nine studies, the increased breast cancer risk associated with carrying a PV was perceived as a certainty of a future diagnosis among some previvors (Dagan and Goldblatt, 2009; DiMillo et al., 2013; Fantini-Hauwel et al., 2025; Glassey et al., 2018b; Haroun et al., 2011; Hyldebrandt et al., 2025; Leonarczyk and Mawn, 2015; Stracke et al., 2022; Tezak et al., 2021). FOCO was commonly described alongside an increased perception of breast cancer risk, as previvors feared cancer could be diagnosed at any time in their lives and often perceived cancer as an ongoing threat (Fantini-Hauwel et al., 2025; Leonarczyk and Mawn, 2015). In nine studies, FOCO was described by breast cancer previvors impacted by a cancer diagnosis in a family member, with many breast cancer previvors certain that their genetic link meant that a cancer diagnosis was unavoidable (Dagan and Goldblatt, 2009; Dean, 2016; Fantini-Hauwel et al., 2025; Glassey et al., 2018b; Haroun et al., 2011; Hyldebrandt et al., 2025; Leonarczyk and Mawn, 2015; Stracke et al., 2022; Tezak et al., 2021). Breast cancer previvors often described themselves as a ticking “time bomb”. This metaphor was used in four studies by previvors describing cancer-related fear arising from the idea that it was only a matter of time before they were diagnosed with breast cancer (Dean, 2016; DiMillo et al., 2013; Glassey et al., 2018b; Leonarczyk and Mawn, 2015). Motherhood also contributed to FOCO, with five studies describing fears of leaving children motherless, children watching their mother die of cancer, or passing the PV to their child(ren) (Dean, 2016; DiMillo et al., 2013; Haroun et al., 2011; Hyldebrandt et al., 2025; Leonarczyk and Mawn, 2015). Interestingly, while the increased perception of breast cancer occurrence was frequently described within studies, no study reported previvors who perceived an increased breast cancer risk but did not experience cancer-related fear.

## Information misinterpretation

Misinterpretation of information in the clinical setting was a driver of FOCO among some breast cancer previvors, both at the time of PV diagnosis and during risk-management decision-making (Glassey et al., 2018b; Leonarczyk and Mawn, 2015). Unclear or conflicting information from clinicians made the selection of risk-management strategies such as bilateral risk-reducing mastectomy or increased breast surveillance challenging, leaving breast cancer previvors feeling isolated and confused (Stracke et al., 2022; Leonarczyk and Mawn, 2015). Conversely, in one study, when previvors felt well-informed about their breast cancer risk, they described feeling comfortable with selecting risk-management strategies. This led to a sense of empowerment, which tempered FOCO (Tezak et al., 2021).

FOCO also arose outside the clinical setting. Internet searches for information about PVs contributed to FOCO, with previvors often misinterpreting search engine results as indicators of breast cancer (Glassey et al., 2018b). Somatic sensations were also attributed to the presence of breast cancer, with the identification of a new breast pain creating FOCO (Fantini-Hauwel et al., 2025).

## Scanxiety

Routine screenings through breast imaging modalities (i.e., mammogram, MRI, or breast ultrasound) is common in the management of an elevated breast cancer risk, serving to monitor and promptly detect breast cancer. In three studies, FOCO was found in breast cancer previvors in the weeks to months before these routine breast screenings and persisted until test results were known (Dean, 2016; DiMillo et al., 2013; Glassey et al., 2018b). The unexpected discovery of breast abnormalities during routine screenings also contributed to FOCO. While previvors often described fear that breast cancer would be diagnosed as a result of routine cancer screening practices (Dean, 2016; DiMillo et al., 2013; Glassey et al., 2018b), no study examined previvors who were aware of their PV but avoided screenings due to fear of breast cancer being diagnosed.

## Discussion

Prior research among previvors has associated the presence of a PV with heightened instances of cancer-related anxiety, worry, and uncertainty regarding an increased risk of developing breast cancer (Dean and Fisher, 2019; Glassey et al., 2018a; Isselhard et al., 2023; Rojas et al., 2019), but cancer-related fear is less understood in the previvor population. This scoping review aimed to synthesize existing literature on FOCO among breast cancer previvors, characterizing how fear of breast cancer manifested in individuals living with a chronically elevated cancer risk due to a PV. FOCO commonly occurred among previvors who perceived that a breast cancer diagnosis was inevitable (Dagan and Goldblatt, 2009; DiMillo et al., 2013; Fantini-Hauwel et al., 2025; Glassey et al., 2018b; Haroun et al., 2011; Hyldebrandt et al., 2025; Leonarczyk and Mawn, 2015; Stracke et al., 2022; Tezak et al., 2021); notably, four studies in this review used the term “time bomb” to describe a certainty that it was only a matter of time until breast cancer was discovered (Dean, 2016; DiMillo et al., 2013; Glassey et al., 2018b; Leonarczyk and Mawn, 2015). This metaphor is found in the previvor literature as well (Dean and Fisher, 2019; Lorke et al., 2021; Torrisi et al., 2025), with some previvors found to conflate risk with the certainty of developing breast cancer (Rutherford et al., 2018) or overestimating their perceived cancer risk (Morgan et al., 2024; Young et al., 2022).

FOCO also manifested as the misinterpretation of information from a variety of sources surrounding breast cancer PVs. Both previvors in this scoping review (Hyldebrandt et al., 2025; Glassey et al., 2018b; Stracke et al., 2022) and in the literature (Dean and Davidson, 2018; Fadda et al., 2020; Torrisi et al., 2024) often turned to clinicians for advice and assistance on education and decision-making to manage an increased breast cancer risk. The information obtained subsequently either helped or hindered the breast cancer previvor's perspective on carrying a PV and set a precedent for seeing the PV as fearful or manageable. While this scoping review noted that information received from the internet contributed to FOCO (Glassey et al., 2018b), in recent years breast cancer previvors have used social media both as a resource to share information and create content, empowering other previvors to proactively manage their increased breast cancer risk (Wellman et al., 2023, 2025). Future research could explore the impact of digital outreach and social media engagement to manage FOCO among breast cancer previvors. Finer (2016) found that these platforms promote adaptive coping and mitigate stress within this population. Lastly, the misinterpretation of a new somatic breast pain as breast cancer contributed to FOCO in this review (Fantini-Hauwel et al., 2025). While not predominant in the previvor literature, breast pain has been found to be a driver of cancer-related fear and excessive self-checking among breast cancer survivors (Lebel et al., 2016; Mutsaers et al., 2016; Soriano et al., 2019).

Scanxiety is a term used to describe the fear, anxiety, and apprehension cancer survivors experience in the period surrounding medical imaging, particularly before results are known (Bui et al., 2021; Derry-Vick et al., 2023; McGinty et al., 2016). This scoping review demonstrates that scanxiety is also a contributing source of FOCO before, during, and after routine breast screening and surveillance (Dean, 2016; DiMillo et al., 2013; Glassey et al., 2018b) in the previvor population. While not widely described in the previvor literature, a 2024 survey identified scanxiety as a primary unmet need among previvors, characterizing the time surrounding routine imaging or biopsy as a period of acute fear while awaiting results (Facing Our Risk of Cancer Empowered, 2024). Recognizing scanxiety in this population represents a critical opportunity for clinical intervention to support the emotional wellbeing of those facing a chronically increased risk of developing cancer.

Several manifestations of FOCO in previvors appear similar to FCR in survivors. Both constructs are characterized by shared triggers, including scanxiety surrounding routine imaging and checking behaviors (Dagan and Goldblatt, 2009; DiMillo et al., 2013; Derry-Vick et al., 2023; Glassey et al., 2018b; Soriano et al., 2019). Additionally, fear of a cancer diagnosis impacts the quality of life in both previvors and survivors (Babadostu and Eyrenci, 2025; Dawson et al., 2016; Dean and Fisher, 2019). However, FOCO also differs from FCR. While FCR is documented as a reactive response to a past cancer diagnosis, FOCO arises from the concern of a future cancer diagnosis. The perception that the presence of a PV could lend itself to an eventual breast cancer diagnosis was the most common manifestation of FOCO across studies in this review (see Table 3). This sense of “ticking time” or being a “time bomb” counting down to a breast cancer diagnosis may be a distinct and clinically significant marker of cancer-related fear in this population.

## Strengths and limitations

This is the first literature review to examine FOCO among breast cancer previvors, a population that chronically experiences an increased risk of developing cancer. From this review, many of the concerns that trigger and describe FOCO appear similar to those of FCR in populations of cancer survivors; thus, understanding how previvors experience FOCO is a strength of this literature review, as it recognizes the potential to address unmet needs and develop interventions to support psychological wellbeing among previvors. An additional strength is the number of qualitative studies exploring FOCO; the use of participants' words to describe fear allowed FOCO to be isolated from similar concepts to establish its unique characteristics. However, this may also serve as a limitation, given that the narrow focus of FOCO may have removed records which used words such as cancer-related distress, anxiety, worry, and uncertainty to describe FOCO.

The choice to limit reports describing FOCO to breast cancer previvors of the female sex also limits generalizability, both to the male sex and to previvors of other types of cancer. Additionally, no validated FOCO-specific measure for breast cancer previvors was identified; this can also be viewed as a limitation. Without the ability to differentiate between the constructs of cancer-related distress, anxiety, worry, uncertainty from fear we may have omitted articles that would otherwise have met inclusion criteria for this scoping review. As a final limitation, this article examined previvors with known PVs and did not examine how FOCO may differ in individuals with a high risk or strong family history but no known PV; this may limit understanding of how FOCO is experienced in high-risk populations.

## Implications

In the literature, FOCO among breast cancer previvors is described more commonly in qualitative research; the absence of quantitative studies may be due to the lack of a standardized measure for cancer-related fear in the previvor population. This might contribute to FOCO being mischaracterized as cancer-related distress, anxiety, worry, and uncertainty, limiting our ability to measure the frequency and severity of this phenomenon. This limitation is supported by a systematic review assessing psychological morbidity in previvors with a BRCA mutation, which examined anxiety, worry, and distress across 45 studies, but did not describe fear of cancer occurrence among previvors (Isselhard et al., 2023). To address this gap, future research should prioritize developing a validated FOCO scale or qualitatively clarifying the links between cancer risk perception and fear in this population. Additional research should also investigate the boundaries of FOCO by examining both resilient previvors who do not experience fear despite their high-risk status, and those who avoid recommended screenings due to FOCO. Moreover, because the existing literature does not define what constitutes a sustainable or manageable level of FOCO, identifying this threshold in future studies could inform both clinical assessment and intervention design targeting FOCO in breast cancer previvors.

## Conclusion

This scoping review examined a critical gap in breast cancer previvorship, fear of cancer occurrence. Limited literature demonstrates that in previvors with a chronically elevated breast cancer risk, FOCO can manifest as a heightened perception of cancer risk, the misinterpretation of information, and scanxiety surrounding routine breast cancer screening practices. To support this population, future research should further explore FOCO in previvors and prioritize developing a standardized instrument to measure cancer-related fear in both clinical and research settings.

## Data Availability

The raw data supporting the conclusions of this article will be made available by the authors, without undue reservation.
